# Matrix Inequalities Based Robust Model Predictive Control for Vehicle Considering Model Uncertainties, External Disturbances, and Time-Varying Delay

**DOI:** 10.3389/fnbot.2020.617293

**Published:** 2021-01-06

**Authors:** Wenjun Liu, Guang Chen, Alois Knoll

**Affiliations:** ^1^Department of Informatics, Technical University of Munich, Munich, Germany; ^2^School of Automotive Studies, Tongji University, Shanghai, China

**Keywords:** robust model predictive control, vehicle dynamic control, matrix inequities, robust positively invariant, model uncertainties, external disturbances, time-varying delay

## Abstract

In this paper, we design a robust model predictive control (MPC) controller for vehicle subjected to bounded model uncertainties, norm-bounded external disturbances and bounded time-varying delay. A Lyapunov-Razumikhin function (LRF) is adopted to ensure that the vehicle system state enters in a robust positively invariant (RPI) set under the control law. A quadratic cost function is selected as the stage cost function, which yields the upper bound of the infinite horizon cost function. A Lyapunov-Krasovskii function (LKF) candidate related to time-varying delay is designed to obtain the upper bound of the infinite horizon cost function and minimize it at each step by using matrix inequalities technology. Then the robust MPC state feedback control law is obtained at each step. Simulation results show that the proposed vehicle dynamic controller can steer vehicle states into a very small region near the reference tracking signal even in the presence of external disturbances, model uncertainties and time-varying delay. The source code can be downloaded on https://github.com/wenjunliu999.

## 1. Introduction

Dynamic control is one of the most crucial tasks for autonomous driving vehicle (Chen et al., 2020). H-infinity output feedback controller (Hu et al., 2016), sliding mode controller (Jiang and Wu, 2018), model predictive control (MPC) (Sun et al., 2019), etc. have been designed for vehicle control. Because MPC has the ability to incorporate soft and hard constraints into the online optimizations in a multivariable control framework, it is widely applied in vehicle control field (Hu et al., 2019).

Vehicle dynamic control performance may be seriously affected by external disturbances resulting from unpredictable environment (Yu et al., 2019). Besides, it is impossible to establish a precise vehicle model when the vehicle is moving (Liu et al., 2019). Therefore, robust model predictive control for vehicle is researched by scholars. Robust feedback MPC (Shamaghdari et al., 2015), tube-based robust MPC (Sakhdari and Azad, 2018), min-max robust MPC (Wang X. et al., 2016), feedback min-max MPC (Liu et al., 2014) and linear matrix inequities (LMIs) or bilinear matrix inequities (BMIs) based robust MPC (Cheng et al., 2020) have been proposed to deal with vehicle control where the vehicle has model uncertainties or external disturbances or both model uncertainties and external disturbances. The optimization problems involving LMIs or BMIs are convex and hence have solvable global optimal solutions and can be solved numerically efficiently and reliably (Duan and Yu, 2013). So, LMIs or BMIs based robust MPC control is widely applied to vehicle control. However, most of existing LMIs or BMIs based robust MPC vehicle control papers only consider the model uncertainties. Few matrix inequalities based robust MPC papers consider both model uncertainties and external disturbances of vehicle.

Moreover, time delay can be frequently seen in vehicle system (Ren et al., 2019) and affects the vehicle dynamic control performance and even threatens the stability and safety of the vehicle system. Some literatures concerned MPC controller for time-delay vehicle system have been addressed (Liu and Li, 2019; Wang et al., 2019). However, most existing papers often assume that the delay is known and fixed (Yu et al., 2018), or do not consider the model uncertainties (Xu et al., 2020) or the external disturbances (Bououden et al., 2016; Nahidi et al., 2019). Therefore, the research on robust MPC controller for vehicle with time-varying delay has not been completely investigated and hence several problems still remain unsolved.

Due to the fact that the influence of the delayed states can cause a violation of the monotonic decrease condition that a standard Lyapunov function obeys, systems affected by delays can not apply the classical Lyapunov theory directly (Gielen et al., 2012a). Generally, there are two types of approaches to deal with time-delay systems, Lyapunov-Razumikhin function (LRF) and Lyapunov-Krasovskii function (LKF) (Teng, 2018). In our paper, we focus on discrete vehicle dynamic control. For discrete-time systems, LKF makes use of an augmentation of the state vector with all delayed states, which yields the applications of classical Lyapunov methods to an augmented system without delay (Teng et al., 2017). LRF is constructed for time-delay system based on a type of small-gain condition (Teel, 1998) and can be considered as a special case of LKF. Compared to LKF, LRF is conservative but its computational complexity is lower than LKF (Gielen et al., 2012b).

In this paper, the aim of dynamic control is to guarantee the vehicle dynamic state tracking performance, where the vehicle dynamic state tracking reference signal is determined by the upper kinematic control and assumed to be known in this paper. To suppress the influence of model uncertainties, external disturbances, and time-varying delay on vehicle dynamic state tracking performance, we design a matrix inequalities based robust MPC controller. It is known that robust positively invariant (RPI) set plays an crucial role in robust MPC, the control law involved in RPI can ensure that when the system state enters the RPI set, it never goes out (Yang and Feng, 2013). A LRF candidate is adopted to guarantee this. Since the infinite horizon cost function can not be optimized online, a cost function with finite terms is usually considered instead. To compute the bound of the infinite horizon cost function, a LKF candidate related to time-varying delay is designed in this paper. Then, the robust MPC state feedback control law is obtained by minimizing the upper bound at each step using matrix inequalities technology.

Different from existing researches, there are two main contributions of this paper. Firstly, the vehicle dynamic model simultaneously considers model uncertainties, external disturbances and the time-varying delay of the vehicle state, which is more in line with actual vehicle operating conditions. Then a robust MPC controller is designed to steer vehicle states into a very small region near the reference tracking signal even in the presence of external disturbances, model uncertainties and time-varying delay. Secondly, compared to the robust controller considering fixed time delay, a delay-range-dependent LKF is designed by using the information of the upper and lower bounds of the time-varying delay and relaxed technique, which also simultaneously takes model uncertainties, external disturbances and the time-varying delay into account.

The rest of this paper is structured as follows. In section 2, two auxiliary lemmas and vehicle model are introduced. In section 3, the proposed robust MPC controller for vehicle subjected to bounded model uncertainties, norm-bounded external disturbances and bounded time-varying delay is designed. In section 4, simulation examples are illustrated to verify the effectiveness of the proposed method. Finally, we conclude in section 5.

*Notations:* ***I*** is an identity matrix with appropriate dimension. diag{⋯ } denotes a block-diagonal matrix, ||·|| denotes 2-norm. ***X*** > 0 and ***X*** ≥ 0 denotes the matrix is a positive definite matrix and positive semi-definite matrix, respectively. The symbol * induces a symmetric structure or a transpose item, e.g., when ***H*** and ***R*** are symmetric matirces, then

$$\left[\begin{array}{cc} H & *\\ S & R \end{array}\right]=\left[\begin{array}{cc} H & S^{\text{T}}\\ S & R \end{array}\right]$$

when the expression has the format ***Q*** + ***S*** + ***S***^T^, we simplify it to ***Q*** + ***S*** + *.

## 2. Preliminaries

In this section, we first introduce two necessary lemmas and then derive the dynamic model of the vehicle with model uncertainties, external disturbances, and time-varying delay.

### 2.1. Auxiliary Lemmas

*Lemma 1:* (Duan and Yu, 2013) Let ***X*** ∈ ***R***^*m* × *n*^, ***Y*** ∈ ***R***^*n* × *m*^, ***Q*** ∈ ***R***^*m* × *m*^. Then

(1) $$\begin{array}{r} Q+XFY+Y^{\text{T}}F^{\text{T}}X^{\text{T}}<0 \end{array}$$

holds for all ***F***^T^***F*** ≤ ***I*** if and only if there exits a positive scalar δ, such that

(2) $$\begin{array}{r} Q+\delta XX^{\text{T}}+\delta^{-1}Y^{\text{T}}Y<0 \end{array}$$

*Lemma 2:* (Jiang and Wang, 2001) If an input-to-state (ISS) Lyapunov function can be found for system ***x***(*k* + 1) = *f*(***x***(*k*), ***p***(*k*)), then the system is ISS.

The definition of ISS-Lyapunov function is as follows.

If a continuous function $V:R^{n}\to R_{\geq 0}$ satisfy the following inequalities, then it is an ISS-Lyapunov function for system ***x***(*k* + 1) = *f*(***x***(*k*), ***p***(*k*)) (Yang and Feng, 2013).

(3) $$\begin{array}{r} \beta_{1}\left(\left||x\left(k\right)|\right|\right)\leq V\left(x\left(k\right)\right)\leq \beta_{2}\left(\left||x\left(k\right)|\right|\right) \end{array}$$

(4) $$\begin{array}{r} V\left(x\left(k+1\right)\right)-V\left(x\left(k\right)\right)\leq -\beta_{3}\left(\left||x\left(k\right)|\right|\right)+\phi \left(\left||p\left(k\right)|\right|\right) \end{array}$$

where β_1_, β_2_, and β_3_ are *K*_∞_-function, ϕ is *K*-function, ***x*** denote system state vector and ***p*** denote external disturbances.

### 2.2. Vehicle Dynamic Model

**Figure 1:**
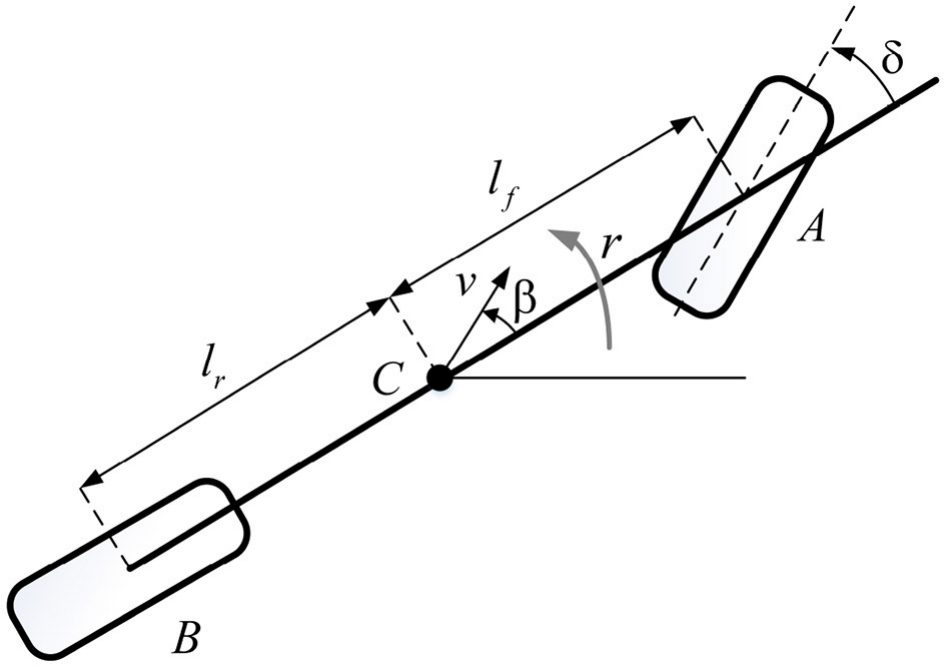
Two-degree-of-freedom bicycle model of vehicle.

An extensively used two-degree-of-freedom bicycle model of vehicle dynamics is adopted in this paper and is shown in Figure 1. This model is based on the assumption of small slip angles, no road gradient or bank angles, no load transfer and no rolling or pitching moment. Only the front wheel is steerable in this case (Elbanhawi et al., 2018). The two front wheels and the two rear wheels are respectively represented by one front wheel A and one rear wheel B. The center of gravity (c.g.) of the vehicle is at point C. The distances of A and B from the c.g. of the vehicle are *l*_*f*_ and *l*_*r*_ respectively. *v* is the velocity at the c.g. of the vehicle, β is the sideslip angle, δ is the steering angle and *r* is the yaw rate. The dynamic model with regard of yaw rate and sideslip angle is adopted in this paper, as presented in (1). The detailed derivation process can be found in Rajamani (2011). *m* is the mass of the vehicle, *C*_α*f*_ and *C*_α*r*_ are the cornering stiffness of the front and rear tires respectively, *I*_*z*_ is yaw moment of inertia. *v*_*x*_ is vehicle longitudinal velocity. Similar to Cho and Huh (2019), a fixed forward velocity assumption is adopted in this paper.

(5) $$\begin{array}{r} \{\begin{array}{c} \dot{\beta }=-r+\frac{C_{\alpha f}}{mv_{x}}\left(\delta -\beta -\frac{l_{f}r}{v_{x}}\right)+\frac{C_{\alpha r}}{mv_{x}}\left(-\beta +\frac{l_{f}r}{v_{x}}\right)\\ ṙ=\frac{l_{f}C_{\alpha f}}{I_{z}}\left(\delta -\beta -\frac{l_{f}r}{v_{x}}\right)-\frac{l_{r}C_{\alpha r}}{I_{z}}\left(-\beta +\frac{l_{f}r}{v_{x}}\right) \end{array} \end{array}$$

Let ***x*** = [β  *r*]^T^, then the state space form of lateral dynamics model can be denoted as follows:

(6) $$\begin{array}{r} \dot{x}=A_{cm}x+B_{cm}\delta \end{array}$$

(7) $$\begin{array}{r} A_{cm}=\left(\begin{array}{cc} -\frac{C_{\alpha f}+C_{\alpha r}}{mv_{x}} & -1+\frac{-l_{f}C_{\alpha f}+l_{r}C_{\alpha r}}{mv_{x}^{2}}\\ \frac{-l_{f}C_{\alpha f}+l_{r}C_{\alpha r}}{I_{z}} & -\frac{l_{f}^{2}C_{\alpha f}+l_{r}^{2}C_{\alpha r}}{I_{z}v_{x}} \end{array}\right) \end{array}$$

(8) $$\begin{array}{r} B_{cm}\;=\;{\left(\begin{array}{cc} \frac{C_{\alpha f}}{mv_{x}} & \frac{l_{f}C_{\alpha f}}{I_{z}} \end{array}\right)}^{\text{T}} \end{array}$$

We discretize the continuous model (6) and consider norm-bounded external disturbances ***p***. ***p*** satisfies ||***p***|| ≤ ρ. Then the system model can be written as follows:

(9) $$\begin{array}{r} x\left(k+1\right)=A_{m}x\left(k\right)+B\delta \left(k\right)+Ep\left(k\right) \end{array}$$

where ***E*** is a constant coefficient matrix, ***A***_*m*_ and ***B*** can be calculated by the Euler method as:

(10) $$\begin{array}{r} A_{m}=e^{A_{cm}T},B=\int_{kT}^{\left(k+1\right)T}e^{A_{cm}\left[\left(k+1\right)T-\eta \right]}B_{cm}d\eta \end{array}$$

where *T* is the sampling interval for the discrete state space model.

Then we consider time-varying delay, the system model can be written as follows:

(11) $$\begin{array}{r} x\left(k+1\right)=Ax\left(k\right)+A_{d}x_{d}\left(k\right)+B\delta \left(k\right)+Ep\left(k\right) \end{array}$$

where ***A*** = α***A***_*m*_, ***A***_*d*_ = (1−α)***A***_*m*_, ***x***_*d*_(*k*) = ***x***(*k* − *d*_*k*_), *d*_*k*_ is a time-varying delay bounded in [*d*_*m*_, *d*_*M*_], *d*_*m*_, *d*_*M*_ are known positive integers, the constant α is the retarded coefficient ranging α ∈ [0, 1], the limits 1 and 0 correspond to no delay term and to a completed delay term, respectively (Jeong and Park, 2005).

It is almost impossible for us to establish a precise model of a vehicle especially when it is moving. So model uncertainties must be considered during the vehicle controller design process.

(12) $$\begin{array}{r} x\left(k+1\right)=\tilde{A}x\left(k\right)+{\tilde{A}}_{d}x_{d}\left(k\right)+\tilde{B}\delta \left(k\right)+Ep\left(k\right) \end{array}$$

where $\tilde{A}=A+MHN_{A}$, ${\tilde{A}}_{d}=A_{d}+MHN_{Ad}$, $\tilde{B}=B+MHN_{B}$, ***M***, ***N***_*A*_, ***N***_*Ad*_ and ***N***_*B*_ are constant matrices, ***H*** is Lebesgue measurable and satisfies ***H***^T^***H*** ≤ ***I***.

## 3. Robust MPC Controller Design Using Matrix Inequalities

In this section, we derive the matrix inequalities involved in the robust MPC controller. MPC is actually an online optimization algorithm. At each iteration time, MPC controller optimizes a cost function and satisfies the corresponding constraints. We first introduce the design of online MPC controller, then we discuss the RPI conditions and develop the computation of a RPI set. Finally, we construct the whole control algorithm.

### 3.1. Online Robust MPC Design

In this paper, the quadratic cost function with infinite length is defined as

(13) $$\begin{array}{r} J_{\infty }\left(k\right)=\overset{\infty }{\underset{i=0}{\sum }}\ell \left(k+i|k\right) \end{array}$$

where ℓ(*k* + *i* ∣ *k*) = ***x***^T^(*k* + *i* ∣ *k*)***Qx***(*k* + *i* ∣ *k*) + **δ**^T^(*k* + *i* ∣ *k*)***R*****δ**(*k* + *i* ∣ *k*) − τ***p***^T^(*k* + *i* ∣ *k*)***p***(*k* + *i* ∣ *k*), ***Q*** and ***R*** are positive definite matrices, τ is a positive constant. ***x***(*k* + *i* ∣ *k*) and **δ**(*k* + *i* ∣ *k*) represent the *i*-th predictive state and control input at time *k* respectively. The choice of *l*(·) is inspired by *H*_∞_ MPC (Mayne et al., 2000) and has already been applied to existing robust MPC research (Yang et al., 2013).

The goal is to find a stabilizing state feedback control **δ**(*k*) = ***Kx***(*k*) for system (12) by using the robust MPC strategy, the online robust MPC optimization can be summarized as:

(14) $$\begin{array}{r} \begin{array}{c} \underset{\delta \left(k+i|k\right)}{\min}\underset{p\left(k+i|k\right)}{\max}J_{\infty }\left(k\right)\\ s.t.\;\left(12\right)\\ -\delta_{\max}\leq \delta \left(k+i|k\right)\leq \delta_{\max}\\ ||p||\leq \gamma \end{array} \end{array}$$

where **δ**_max_ is the input constraint.

Considering *J*_∞_(*k*) can not be minimized directly in reality, an upper bound of the infinite horizon cost function is minimized instead. To find an upper bound of *J*_∞_(*k*), a LKF candidate related to time-varying delay is designed as follows:

(15) $$\begin{array}{r} V\left(x\left(k\right)\right)=V_{1}\left(x\left(k\right)\right)+V_{2}\left(x\left(k\right)\right)+V_{3}\left(x\left(k\right)\right) \end{array}$$

(16) $$\begin{array}{r} V_{1}\left(x\left(k\right)\right)=x^{\text{T}}\left(k\right)Px\left(k\right) \end{array}$$

(17) $$\begin{array}{r} V_{2}\left(x\left(k\right)\right)=\overset{k-1}{\underset{i=k-d_{k}}{\sum }}x^{\text{T}}\left(i\right)P_{d}x\left(i\right) \end{array}$$

(18) $$\begin{array}{r} V_{3}\left(x\left(k\right)\right)=\overset{-d_{m}}{\underset{j=-d_{M}+1}{\sum }}\overset{k-1}{\underset{i=k+j}{\sum }}x^{\text{T}}\left(i\right)P_{d}x\left(i\right) \end{array}$$

where ***P*** = ***P***^T^ > 0, $P_{d}=P_{d}^{\text{T}}>0$. And suppose the following inequality is satisfied.

(19) $$\begin{array}{r} V\left(x\left(k+i+1|k\right)\right)-V\left(x\left(k+i|k\right)\right)\leq -l\left(k+i|k\right) \end{array}$$

As shown in Kothare et al. (1996), for the robust performance objective function to be finite, we must have ***x***(∞∣*k*) = 0 and hence *V*(***x***(∞∣*k*)) = 0. Summing (19) from *i* = 0 to *i* = ∞, we get

(20) $$\begin{array}{r} J_{\infty }\left(k\right)\leq V\left(x\left(k|k\right)\right)\leq \xi \left(k\right) \end{array}$$

where ξ(*k*) > 0 is the upper bound of *J*_∞_(*k*).

*Theorem 1:* If there exit matrices with appropriate dimension ***X*** = ***X***^T^ > 0, $X_{d}=X_{d}^{\text{T}}>0$, a general matrix ***Y*** and a scalar η > 0, then the following LMIs can be derived to guarantee (19) i.e., to guarantee *V*(***x***(*k* ∣ *k*)) is the upper bound of *J*_∞_(*k*).

(21) $$\begin{array}{r} \left[\begin{array}{ccccccccc} -X & * & * & * & * & * & * & * & *\\ 0 & -X_{d} & * & * & * & * & * & * & *\\ 0 & 0 & -\tau \xi I & * & * & * & * & * & *\\ AX+BY & A_{d}X_{d} & \xi E & -X & * & * & * & * & *\\ X & 0 & 0 & 0 & -{\left(d_{s}+1\right)}^{-1}X_{d} & * & * & * & *\\ QX & 0 & 0 & 0 & 0 & -\xi Q & * & * & *\\ RY & 0 & 0 & 0 & 0 & 0 & -\xi R & * & *\\ N_{A}X+N_{B}Y & N_{Ad}X_{d} & 0 & 0 & 0 & 0 & 0 & -\eta I & *\\ 0 & 0 & 0 & \eta M^{\text{T}} & 0 & 0 & 0 & 0 & -\eta I \end{array}\right]<0 \end{array}$$

where *d*_*s*_ = *d*_*M*_ − *d*_*m*_.

*Proof:* See Appendix A.

Equation (21) can guarantee ξ(*k*) is the upper bound of *J*_∞_(*k*), then we minimize it.

*Theorem 2:* ξ(*k*) can be minimized using the following LMIs.

(22) $$\begin{array}{r} \left[\begin{array}{cc} 1 & *\\ \zeta_{3}\left(k\right) & \Lambda_{3} \end{array}\right]\geq 0 \end{array}$$

where $\zeta_{3}\left(k\right)=[x^{\text{T}}\left(k\right),x^{\text{T}}\left(k-1\right),\cdots \;,x^{\text{T}}\left(k-d_{m}\right),x^{\text{T}}$ $\left(k-d_{m}-1\right),\cdots \;,x^{\text{T}}\left(k-d_{M}\right){]}^{\text{T}},\Lambda_{3}=\mathrm{diag}(P,\frac{P_{d}}{d_{s}+1},\frac{P_{d}}{d_{s}},\cdots \;,P_{d})$.

*Proof:*

(23) $$\begin{array}{l} V_{2}\left(x\left(k\right)\right)=\overset{k-1}{\underset{i=k-d_{k}}{\sum }}x^{\text{T}}\left(i\right)P_{d}x\left(i\right)\leq \overset{k-1}{\underset{i=k-d_{M}}{\sum }}x^{\text{T}}\left(i\right)P_{d}x\left(i\right)\\ =\zeta_{1}^{\text{T}}\left(k-1\right)\Lambda_{1}\zeta_{1}\left(k-1\right) \end{array}$$

where $\zeta_{1}\left(k-1\right)={\left[x^{\text{T}}\left(k-1\right),\cdots \;,x^{\text{T}}\left(k-d_{M}\right)\right]}^{\text{T}},\Lambda_{1}=\mathrm{diag}\left(P_{d},\cdots \;,P_{d}\right)$.

(24) $$\begin{array}{r} V_{3}\left(x\left(k\right)\right)=\overset{-d_{m}}{\underset{j=-d_{M}+1}{\sum }}\overset{k-1}{\underset{i=k+j}{\sum }}x^{\text{T}}\left(i\right)P_{d}x\left(i\right)=\zeta_{2}^{T}\left(k-1\right)\Lambda_{2}\zeta_{2}\left(k-1\right) \end{array}$$

where $\zeta_{2}\left(k-1\right)=[x^{\text{T}}\left(k-1\right),\cdots \;,x^{\text{T}}\left(k-d_{m}\right),x^{\text{T}}\left(k-d_{m}-1\right),$ $\cdots \;,x^{\text{T}}\left(k-d_{M}+1\right){]}^{\text{T}}$, **Λ**_2_ = diag(*d*_*s*_***P***_*d*_, (*d*_*s*_−1)***P***_*d*_, ⋯ , ***P***_*d*_).

Summing (16), (23), (24), we can conclude that

(25) $$\begin{array}{r} V\left(x\left(k\right)\right)=\zeta_{3}^{\text{T}}\left(k\right)\Lambda_{3}\zeta_{3}\left(k\right) \end{array}$$

Substituting $X=\xi \left(k\right)P^{-1},X_{d}=\xi \left(k\right)P_{d}^{-1}$, then by using Schur complement, (22) can be obtained.

### 3.2. Robust Positively Invariant Set Computation

In this section, we design a LRF to compute RPI set. The RPI set is defined as follows:

(26) $$\begin{array}{r} \Omega =\left\{\left\{x,x_{d}\right\}|\max\left\{x^{\text{T}}Px,x_{d}^{\text{T}}P_{d}x_{d}\right\}\leq \xi \right\} \end{array}$$

*Lemma 3:* Consider system (12), the set **Ω** is a RPI if there exists a positive scalar γ ∈ (0, 1) such that:

(27) $$\begin{array}{r} \frac{1}{\xi }x^{\text{T}}\left(k+1\right)Px\left(k+1\right)-\frac{1-\lambda }{\xi }\max\left\{x^{\text{T}}Px,x_{d}^{\text{T}}Px_{d}\right\}-\frac{\lambda }{\rho^{2}}p^{\text{T}}p\leq 0 \end{array}$$

Proof:

According to (27), $\max\left\{x^{\text{T}}Px,x_{d}^{\text{T}}P_{d}x_{d}\right\}\leq \xi$ and ***P***^T^***P*** ≤ ρ^2^, we have:

(28) $$\begin{array}{l} \frac{1}{\xi }x^{\text{T}}\left(k+1\right)Px\left(k+1\right)\leq \frac{1-\lambda }{\xi }\max\left\{x^{\text{T}}Px,x_{d}^{\text{T}}Px_{d}\right\}+\frac{\lambda }{\rho^{2}}p^{\text{T}}p\\ \;\leq \left(1-\lambda \right)+\lambda =1 \end{array}$$

So we can get ***x***^T^(*k* + 1)***Px***(*k* + 1) ≤ ξ, that means ***x***(*k* + 1) is still in the RPI set.

*Theorem 3:* If there exists a positive definite matrix ***X***, a general matrix ***Y*** and positive scalars σ and λ, such that the following BMIs and LMIs hold.

(29) $$\begin{array}{r} \left[\begin{array}{cccccc} \gamma \left(\lambda -1\right)X & * & * & * & * & *\\ 0 & \gamma_{d}\left(\lambda -1\right)X & * & * & * & *\\ 0 & 0 & -\frac{\lambda }{\rho^{2}}I & * & * & *\\ AX+BY & A_{d}X & E & -X & * & *\\ N_{A}X+N_{B}Y & N_{Ad}X & 0 & 0 & -\sigma I & *\\ \sigma M^{\text{T}} & 0 & 0 & 0 & 0 & -\sigma I \end{array}\right]\leq 0 \end{array}$$

(30) $$\begin{array}{r} \left[\begin{array}{cc} Z & *\\ Y^{\text{T}} & X \end{array}\right]\geq 0 \end{array}$$

where γ + γ_*d*_ = 1, $Z_{tt}\leq \delta_{t,max}^{2}$, *Z*_*tt*_ is the *t*-th diagonal element of matrix ***Z***.

*Proof:* See Appendix B.

### 3.3. Online Robust MPC Algorithm

We summarize the robust MPC algorithm based on the above-mentioned results. The implementation of the robust controller is summarized as follows.

(31) $$\begin{array}{r} \begin{array}{c} \underset{\xi ,X,X_{d},Y,\lambda ,\eta ,\sigma }{\min}\xi \\ s.t.\left(21\right),\left(22\right),\left(29\right),\left(30\right) \end{array} \end{array}$$

Then the control input at time *k* can be computed **δ**(*k*) = ***Kx***(*k*). Note that (29) is BMIs, in our paper, to reduce the computation burden, we only calculate the optimal λ^*^ of λ at the initial time, and replace λ with λ^*^ in subsequent iterations.

*Theorem 4:* If the optimization problem (31) is solvable for vehicle system (12) at the initial time, then the vehicle system (12) is ISS with regard to the external disturbances.

*Proof:* See Appendix C.

*Remark:* In this paper, two Lyapunov functions (LKF and LRF) are used at the same time. But LRF is only used to ensure that the system enters RPI, which provides a tighter constraint. As shown in Gielen et al. (2012a), the existence of a LRF is a sufficient condition for the existence of a LKF.

## 4. Simulation and Analysis

In this section, we conduct three simulation scenarios to validate the effectiveness of the designed robust MPC controller. The vehicle model parameters *m*, *I*_*z*_, *l*_*f*_, *l*_*r*_, *C*_α*f*_, *C*_α*r*_, *I*_*z*_ and *v*_*x*_ of the simulation vehicle are 1, 000kg, 1, 650kg/m^2^, 1.0m, 1.6m, 3, 000N/rad, 3, 000N/rad and 10m/s, respectively.

**Figure 2:**
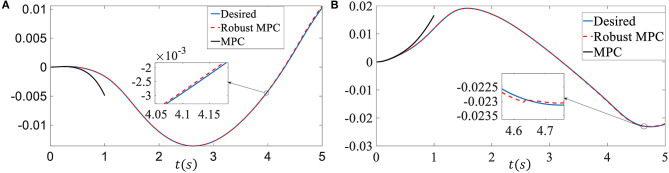
Control performance comparison of the first simulation. **(A)** Sideslip angle. **(B)** Yaw rate.

In the first simulation, we assume there is no time-varying delay, 2% model uncertainties are added, i.e., ***N***_*A*_ = 0.02***A***, ***N***_*B*_ = 0.02***B***. ***M*** is an identity matrix, ***E*** = [0.01 0.1]^T^, ***Q*** is diag{5, 5}, ***R*** is 1, τ is 1. The external disturbances ***p*** and are given by ***p***(*k*) = 1 × 10^−7^ sin(*k*), ***H***(*k*) = sin(*k*) respectively (At each time step, the magnitude of the increment of β and *r* are 1 × 10^−7^ and 1 × 10^−6^ respectively). The input constraint is ||**δ**|| ≤ 0.5 rad. The sampling time is 0.01 s. Under the same conditions, we compared the performance of the proposed robust MPC with the traditional MPC algorithm (Elbanhawi et al., 2018) on the reference signal tracking problem. Figure 2A is the sideslip angle tracking performance comparison between the proposed robust MPC and traditional MPC. Figure 2B is the yaw rate tracking performance comparison between the proposed robust MPC and traditional MPC. It can be easily seen that the proposed robust MPC has a good tracking performance even there exist model uncertainties and external disturbances. However, traditional MPC controller becomes unstable and can not execute the control. Please note that the performance of MPC control is seriously degraded in the presence of external disturbances and model uncertainties. To observe the performance comparison of the MPC controller and the proposed robust MPC controller in the same figure more clearly, we only show the MPC controller tracking trajectory for 1s in the figure. Otherwise, the tracking trajectory generated by the robust MPC controller and the desired tracking trajectory will look like a straight line because of the scale.

**Figure 3:**
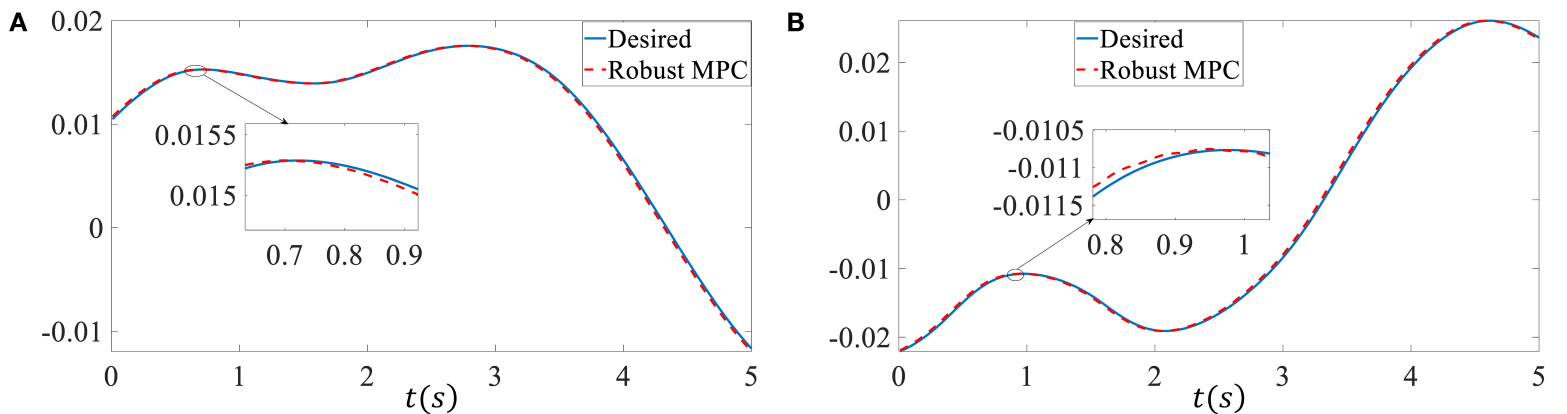
Control performance of the second simulation. **(A)** Sideslip angle. **(B)** Yaw rate.

In the second simulation, we assume the vehicle system has time-varying delay, the retarded coefficient α is 0.8, *d*_*m*_ is 1, *d*_*M*_ is 3, γ is 0.8, γ_*d*_ is 0.2. 5% model uncertainties are added, i.e., ***N***_*A*_ = 0.05***A***, ***N***_*B*_ = 0.05***B***, ***N***_*Ad*_ = 0.05***A***_*d*_. ***M*** is an identity matrix, ***E*** = [0.01 0.1]^T^, ***Q*** is diag{5, 5}, ***R*** is 1, τ is 1. The external disturbances ***p*** and ***H*** are given by ***p***(*k*) = 1 × 10^−3^ sin(*k*), ***H***(*k*) = sin(*k*) respectively. The input constraint is ||**δ**|| ≤ 0.5 rad. The sampling time is 0.01 s. Figure 3 shows the proposed controller can track the reference well when there exist model uncertainties, external disturbances, and time-varying delay. The root mean square error (RMSE) of the sideslip angle tracking error is 7.8719 × 10^−6^, the RMSE of the yaw rate tracking error is 7.996 × 10^−5^.

**Figure 4:**
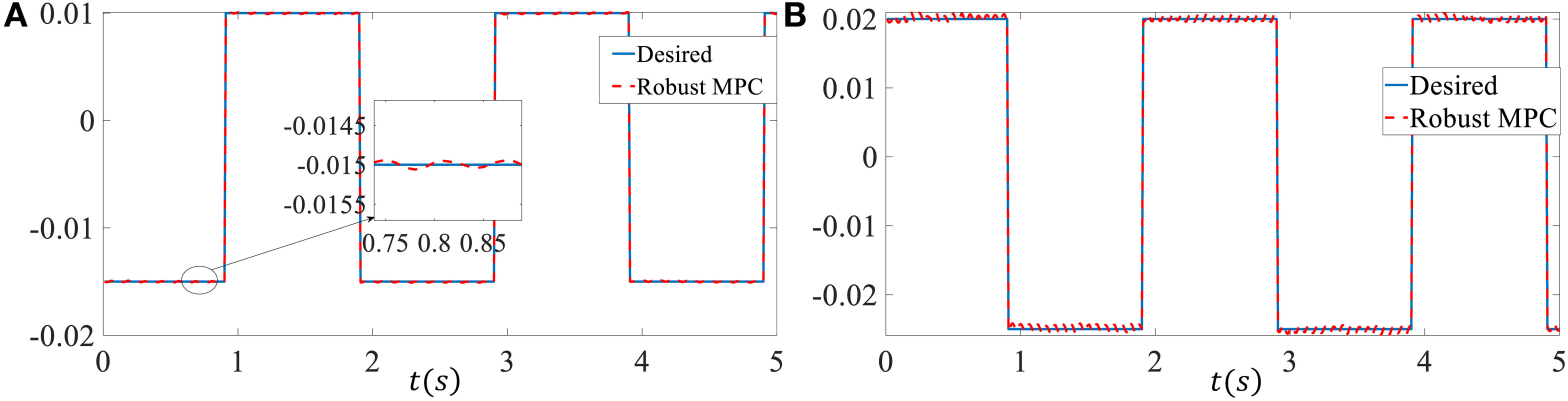
Control performance of the third simulation. **(A)** Sideslip angle. **(B)** Yaw rate.

In order to more convincingly verify the effectiveness of the proposed controller, we chose square wave reference signals as the tracking signal in the third simulation. The upper bound of delay is set as *d*_*M*_ = 10, the external disturbances ***p*** is set as ***p***(*k*) = 5 × 10^−3^ sin(*k*). Other parameters are the same as in simulation 2. As shown in Figure 4, despite the time-varying delay, model uncertainties and external disturbances, the proposed controller can still track the challenging reference signal well. The system states come into a very small region near the neighborhood of the reference tracking trajectory in the presence of a bigger external disturbances. The RMSE of the sideslip angle tracking error is 6.3077 × 10^−5^, the RMSE of the yaw rate tracking error is 4.8308 × 10^−4^.

**Figure 5:**
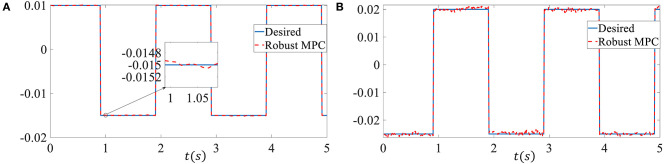
Control performance of the fourth simulation. **(A)** Sideslip angle. **(B)** Yaw rate.

To further verify the robustness of the proposed controller, we consider 10% model uncertainties and the external disturbances ***p*** are set as ***p*** = 5 × 10^−3^ sin(0.1π*k*), where the frequency of the disturbance refers to Wang N. et al. (2016). Other parameters are the same as in simulation 3. The square wave reference signals are also selected as the tracking signal in the this simulation. The performance of the proposed robust MPC controller is shown in Figure 5. The yaw rate tracking performance is a bit worse but it is still stable in the neighborhood of the reference tracking trajectory in the presence of bigger model uncertainties and bigger disturbance frequency.

## 5. Conclusion

In this paper, we design a robust MPC controller for vehicle system with model uncertainties, external disturbances, and time-varying delay. A LKF candidate related to time-varying delay is constructed to derive an upper bound of the cost function. A LRF is designed to compute the RPI. The computation of RPI and upper bound of the cost function, input constraint and the minimization problem are expressed as LMIs or BMIs by using matrix technology. Then the MPC state feedback law is calculated in terms of LMIs/BMIs. Simulation results show that the proposed controller is effective.

## Data Availability Statement

The original contributions presented in the study are included in the article/Supplementary Material, further inquiries can be directed to the corresponding author/s.

## Author Contributions

WL: methodology, software, data curation, and writing original draft. GC: methodology and data curation. AK: conceptualization, supervision, and writing—review. All authors contributed to the article and approved the submitted version.

## Conflict of Interest

The authors declare that the research was conducted in the absence of any commercial or financial relationships that could be construed as a potential conflict of interest.
