# Supplementary material for: Matrix Inequalities Based Robust Model Predictive Control for Vehicle Considering Model Uncertainties, External Disturbances, and Time-Varying Delay
Source: Front Neurorobot. 2021 Jan 6;14:617293. doi: 10.3389/fnbot.2020.617293 (PMC7815701; doi:10.3389/fnbot.2020.617293)
Supplement: Supplementary file 1 [file Presentation_1.pdf]

Supplementary Material

APPENDIX A

Proof: Substituting $\boldsymbol{\delta}(k) = \boldsymbol{K}\boldsymbol{x}(k)$ to (12), the closed-loop vehicle dynamic system can be obtained as:

$$\boldsymbol{x}(k+1) = \boldsymbol{A}_C(k)\boldsymbol{x}(k) + \boldsymbol{A}_D(k)\boldsymbol{x}_d(k) + \boldsymbol{E}\boldsymbol{p}(k) \tag{S1}$$

where $\boldsymbol{A}_C = \boldsymbol{A} + \boldsymbol{B}\boldsymbol{K} + \boldsymbol{M}\boldsymbol{H}\left(\boldsymbol{N}_A + \boldsymbol{N}_B\boldsymbol{K}\right), \boldsymbol{A}_D = \boldsymbol{A}_d + \boldsymbol{M}\boldsymbol{H}\boldsymbol{N}_{Ad}$

$$\begin{aligned}
&\Delta V_1(\boldsymbol{x}(k)) = \boldsymbol{x}^{\mathrm{T}}(k+1 \mid k)\boldsymbol{P}\boldsymbol{x}(k+1 \mid k) - \boldsymbol{x}^{\mathrm{T}}(k \mid k)\boldsymbol{P}\boldsymbol{x}(k \mid k) \\
&= \left[\boldsymbol{A}_C\boldsymbol{x}(k) + \boldsymbol{A}_D\boldsymbol{x}_d(k)) + \boldsymbol{E}\boldsymbol{p}(k)\right]^{\mathrm{T}} \boldsymbol{P}\left[\boldsymbol{A}_C\boldsymbol{x}(k) + \boldsymbol{A}_D\boldsymbol{x}_d(k) + \boldsymbol{E}\boldsymbol{p}(k)\right] - \boldsymbol{x}^{\mathrm{T}}(k)\boldsymbol{P}\boldsymbol{x}(k) \\
&= \begin{bmatrix} \boldsymbol{x}^{\mathrm{T}}(k) & \boldsymbol{x}_d^{\mathrm{T}}(k) & \boldsymbol{p}(k)^{\mathrm{T}} \end{bmatrix} \begin{bmatrix} \boldsymbol{A}_C^{\mathrm{T}} \\ \boldsymbol{A}_D^{\mathrm{T}} \\ \boldsymbol{E}^{\mathrm{T}} \end{bmatrix} \boldsymbol{P} \begin{bmatrix} \boldsymbol{A}_C & \boldsymbol{A}_D & \boldsymbol{E} \end{bmatrix} \begin{bmatrix} \boldsymbol{x}(k) \\ \boldsymbol{x}_d(k) \\ \boldsymbol{p}(k) \end{bmatrix} - \boldsymbol{x}^{\mathrm{T}}(k)\boldsymbol{P}\boldsymbol{x}(k) \\
&= \boldsymbol{\zeta}^{\mathrm{T}}(k)\boldsymbol{\Pi}_1^{\mathrm{T}}\boldsymbol{P}\boldsymbol{\Pi}_1\boldsymbol{\zeta}(k) - \boldsymbol{x}^{\mathrm{T}}(k)\boldsymbol{P}\boldsymbol{x}(k)
\end{aligned} \tag{S2}$$

where $\boldsymbol{\zeta}(k) = \begin{bmatrix} \boldsymbol{x}(k) & \boldsymbol{x}_d(k) & \boldsymbol{p} \end{bmatrix}^{\mathrm{T}}, \boldsymbol{\Pi}_1 = \begin{bmatrix} \boldsymbol{A}_C & \boldsymbol{A}_D & \boldsymbol{E} \end{bmatrix}$

$$\begin{aligned}
&\Delta V_2(\boldsymbol{x}(k)) = \sum_{i=k+1-d_{k+1}}^{k} \ell(\boldsymbol{x}^{\mathrm{T}}(i)\boldsymbol{P}_d\boldsymbol{x}(i)) - \sum_{i=k-d_k}^{k-1} \boldsymbol{x}^{\mathrm{T}}(i)\boldsymbol{P}_d\boldsymbol{x}(i) \\
&= \boldsymbol{x}^{\mathrm{T}}(k)\boldsymbol{P}_d\boldsymbol{x}(k) - \boldsymbol{x}_d^{\mathrm{T}}(k)\boldsymbol{P}_d\boldsymbol{x}_d(k) + \sum_{i=k+1-d_{k+1}}^{k-1} \boldsymbol{x}^{\mathrm{T}}(i)\boldsymbol{P}_d\boldsymbol{x}(i) - \sum_{i=k-d_k+1}^{k-1} \boldsymbol{x}^{\mathrm{T}}(i)\boldsymbol{P}_d\boldsymbol{x}(i) \\
&\leq \boldsymbol{x}^{\mathrm{T}}(k)\boldsymbol{P}_d\boldsymbol{x}(k) - \boldsymbol{x}_d^{\mathrm{T}}(k)\boldsymbol{P}_d\boldsymbol{x}_d(k) + \sum_{i=k+1-d_M}^{k-1} \boldsymbol{x}^{\mathrm{T}}(i)\boldsymbol{P}_d\boldsymbol{x}(i) - \sum_{i=k-d_m+1}^{k-1} \boldsymbol{x}^{\mathrm{T}}(i)\boldsymbol{P}_d\boldsymbol{x}(i) \\
&= \boldsymbol{x}^{\mathrm{T}}(k)\boldsymbol{P}_d\boldsymbol{x}(k) - \boldsymbol{x}_d^{\mathrm{T}}(k)\boldsymbol{P}_d\boldsymbol{x}_d(k) + \sum_{i=k+1-d_M}^{k-d_m} \boldsymbol{x}^{\mathrm{T}}(i)\boldsymbol{P}_d\boldsymbol{x}(i)
\end{aligned} \tag{S3}$$

$$\begin{aligned}
&\Delta V_3(\boldsymbol{x}(k)) = \sum_{j=-d_M+1}^{-d_m} \left[\sum_{i=k+j+1}^{k} \boldsymbol{x}^{\mathrm{T}}(i)\boldsymbol{P}_d\boldsymbol{x}(i) - \sum_{i=k+j}^{k-1} \boldsymbol{x}^{\mathrm{T}}(i)\boldsymbol{P}_d\boldsymbol{x}(i)\right] \\
&= \sum_{j=-d_M+1}^{-d_m} \left[\boldsymbol{x}^{\mathrm{T}}(k)\boldsymbol{P}_d\boldsymbol{x}(k) - \boldsymbol{x}^{\mathrm{T}}(k+j)\boldsymbol{P}_d\boldsymbol{x}(k+j)\right] \\
&= d_s\boldsymbol{x}^{\mathrm{T}}(k)\boldsymbol{P}_d\boldsymbol{x}(k) - \sum_{i=k-d_M+1}^{k-d_m} \boldsymbol{x}^{\mathrm{T}}(i)\boldsymbol{P}_d\boldsymbol{x}(i)
\end{aligned} \tag{S4}$$

Combining (S2), (S3) and (S5), we can get:

$$\begin{aligned}
&\Delta V(\boldsymbol{x}(k)) = \Delta V_1(\boldsymbol{x}(k)) + \Delta V_2(\boldsymbol{x}(k)) + \Delta V_3(\boldsymbol{x}(k)) \\
&\leq \boldsymbol{\zeta}^{\mathrm{T}}(k)\boldsymbol{\Pi}_1^{\mathrm{T}}\boldsymbol{P}\boldsymbol{\Pi}_1\boldsymbol{\zeta}(k) - \boldsymbol{x}^{\mathrm{T}}(k)\boldsymbol{P}\boldsymbol{x}(k) \\
&+\boldsymbol{x}^{\mathrm{T}}(k)\boldsymbol{P}_d\boldsymbol{x}(k) - \boldsymbol{x}_d^{\mathrm{T}}(k)\boldsymbol{P}_d\boldsymbol{x}_d(k) + d_s\boldsymbol{x}^{\mathrm{T}}(k)\boldsymbol{P}_d\boldsymbol{x}(k) \\
&= \boldsymbol{\zeta}^{\mathrm{T}}(k)\left(\boldsymbol{\Pi}_1^{\mathrm{T}}\boldsymbol{P}\boldsymbol{\Pi}_1 + \boldsymbol{\Theta}_1\right)\boldsymbol{\zeta}(k)
\end{aligned} \tag{S5}$$

where $\boldsymbol{\Theta}_1 = \mathrm{diag}\left(\left(d_s+1\right)\boldsymbol{P}_d - \boldsymbol{P}, -\boldsymbol{P}_d, 0\right)$.

The predicted stage cost can be rewritten as:

$$\begin{aligned}
&l(k+i \mid k) = \boldsymbol{x}^{\mathrm{T}}(k+i \mid k)\boldsymbol{Q}\boldsymbol{x}(k+i \mid k) + \boldsymbol{\delta}^{\mathrm{T}}(k+i \mid k)\boldsymbol{R}\boldsymbol{\delta}(k+i \mid k) - \tau\boldsymbol{p}^{\mathrm{T}}(k+i \mid k)\boldsymbol{p}(k+i \mid k) \\
&= \left[\begin{array}{ccc} \boldsymbol{x}^{\mathrm{T}}(k) & \boldsymbol{x}_d^{\mathrm{T}}(k) & \boldsymbol{p}(k)^{\mathrm{T}} \end{array}\right] \mathrm{diag}\left(\boldsymbol{Q} + \boldsymbol{K}^{\mathrm{T}}\boldsymbol{R}\boldsymbol{K}, 0, -\tau\boldsymbol{I}\right) \left[\begin{array}{c} \boldsymbol{x}(k) \\ \boldsymbol{x}_d(k) \\ \boldsymbol{p}(k) \end{array}\right] \\
&= \boldsymbol{\zeta}^{T}(k)\boldsymbol{\Theta}_2\boldsymbol{\zeta}(k)
\end{aligned} \tag{S6}$$

where $\boldsymbol{\Theta}_2 = \mathrm{diag}\left(\boldsymbol{Q} + \boldsymbol{K}^{T}\boldsymbol{R}\boldsymbol{K}, 0, -\tau\boldsymbol{I}\right)$

If $\boldsymbol{\zeta}^{\mathrm{T}}(k)\left(\boldsymbol{\Pi}_1^{\mathrm{T}}\boldsymbol{P}\boldsymbol{\Pi}_1 + \boldsymbol{\Theta}_1\right)\boldsymbol{\zeta}(k) \leq -\boldsymbol{\zeta}^{\mathrm{T}}(k)\boldsymbol{\Theta}_2\boldsymbol{\zeta}(k)$, i.e. $\boldsymbol{\Pi}_1^{\mathrm{T}}\boldsymbol{P}\boldsymbol{\Pi}_1 + \boldsymbol{\Theta}_1 \leq -\boldsymbol{\Theta}_2$ then $\Delta V(\boldsymbol{x}(k)) \leq -l(k+i \mid k)$ can be guaranteed, i.e. $V(\boldsymbol{x}(k \mid k))$ is the upper bound of the infinite cost function.

Substituting $\boldsymbol{\Pi}_1 = \left[\begin{array}{ccc} \boldsymbol{A}_C & \boldsymbol{A}_D & \boldsymbol{E} \end{array}\right]$, $\boldsymbol{\Theta}_1 = \mathrm{diag}\left(\left(d_s+1\right)\boldsymbol{P}_d - \boldsymbol{P}, -\boldsymbol{P}_d, 0\right)$ and $\boldsymbol{\Theta}_2 = \mathrm{diag}\left(\boldsymbol{Q} + \boldsymbol{K}^{\mathrm{T}}\boldsymbol{R}\boldsymbol{K}, 0, -\tau\boldsymbol{I}\right)$ into $\boldsymbol{\Pi}_1^{\mathrm{T}}\boldsymbol{P}\boldsymbol{\Pi}_1 + \boldsymbol{\Theta}_1 \leq -\boldsymbol{\Theta}_2$, we can get:

$$\left[\begin{array}{ccc} \left(d_s+1\right)\boldsymbol{P}_d - \boldsymbol{P} + \boldsymbol{Q} + \boldsymbol{K}^{\mathrm{T}}\boldsymbol{R}\boldsymbol{K} & 0 & 0 \\ 0 & -\boldsymbol{P}_d & 0 \\ 0 & 0 & -\tau\boldsymbol{I} \end{array}\right] + \left[\begin{array}{c} \boldsymbol{A}_C^{\mathrm{T}} \\ \boldsymbol{A}_D^{\mathrm{T}} \\ \boldsymbol{E}^{\mathrm{T}} \end{array}\right]\boldsymbol{P}\left[\begin{array}{ccc} \boldsymbol{A}_C & \boldsymbol{A}\boldsymbol{D} & \boldsymbol{E} \end{array}\right] \leq 0 \tag{S7}$$

Substituting $\boldsymbol{X}_d = \xi\boldsymbol{P}_d^{-1}$ and $\boldsymbol{X} = \xi\boldsymbol{P}^{-1}$ to the above inequality, then using Schur complement, we can get:

$$\left[\begin{array}{ccccccc}
-\xi\boldsymbol{X}^{-1} & * & * & * & * & * & * \\
0 & -\xi\boldsymbol{X}_d^{-1} & * & * & * & * & * \\
0 & 0 & -\tau\boldsymbol{I} & * & * & * & * \\
\boldsymbol{A}_C & \boldsymbol{A}_D & \boldsymbol{E} & -\xi^{-1}\boldsymbol{X} & * & * & * \\
\boldsymbol{I} & 0 & 0 & 0 & -\left(d_s+1\right)^{-1}\xi^{-1}\boldsymbol{X}_d & * & * \\
\boldsymbol{Q} & 0 & 0 & 0 & 0 & -\boldsymbol{Q} & * \\
\boldsymbol{R}\boldsymbol{K} & 0 & 0 & 0 & 0 & 0 & -\boldsymbol{R}
\end{array}\right] < 0 \tag{S8}$$

Multiplying $\mathrm{diag}\left\{\xi^{-1/2}, \xi^{-1/2}, \xi^{1/2}, \xi^{1/2}, \xi^{1/2}, \xi^{1/2}, \xi^{1/2}\right\}$ on the both sides of the above inequality.

$$\left[\begin{array}{ccccccc}
-\boldsymbol{X}^{-1} & * & * & * & * & * & * \\
0 & -\boldsymbol{X}_d^{-1} & * & * & * & * & * \\
0 & 0 & -\xi\tau\boldsymbol{I} & * & * & * & * \\
\boldsymbol{A}_C & \boldsymbol{A}_D & \xi\boldsymbol{E} & -\boldsymbol{X} & * & * & * \\
\boldsymbol{I} & 0 & 0 & 0 & -\left(d_s+1\right)^{-1}\boldsymbol{X}_d & * & * \\
\boldsymbol{Q} & 0 & 0 & 0 & 0 & -\xi\boldsymbol{Q} & * \\
\boldsymbol{R}\boldsymbol{K} & 0 & 0 & 0 & 0 & 0 & -\xi\boldsymbol{R}
\end{array}\right] \tag{S9}$$

Multiplying $\mathrm{diag}\left\{\boldsymbol{X}, \boldsymbol{X}_d, \boldsymbol{I}, \boldsymbol{I}, \boldsymbol{I}, \boldsymbol{I}, \boldsymbol{I}\right\}$ on the both sides of the above inequality, we can get:

$$\begin{bmatrix} -\boldsymbol{X} & * & * & * & * & * & * \\ 0 & -\boldsymbol{X}_d & * & * & * & * & * \\ 0 & 0 & -\xi\tau\boldsymbol{I} & * & * & * & * \\ \boldsymbol{A}_C\boldsymbol{X} & \boldsymbol{A}_D\boldsymbol{X}_d & \xi\boldsymbol{E} & -\boldsymbol{X} & * & * & * \\ \boldsymbol{X} & 0 & 0 & 0 & -\left(d_s+1\right)^{-1}\boldsymbol{X}_d & * & * \\ \boldsymbol{Q}\boldsymbol{X} & 0 & 0 & 0 & 0 & -\xi\boldsymbol{Q} & * \\ \boldsymbol{R}\boldsymbol{K}\boldsymbol{X} & 0 & 0 & 0 & 0 & 0 & -\xi\boldsymbol{R} \end{bmatrix} \tag{S10}$$

Substituting $\boldsymbol{A}_C$, $\boldsymbol{A}_D$ and $\boldsymbol{Y} = \boldsymbol{K}\boldsymbol{X}$ to the above inequality. Then according to Lemma 1, the above inequality holds if and only if there exists some positive scalar η, such that:

$$\begin{bmatrix} -\boldsymbol{X} & * & * & * & * & * & * & * & * \\ 0 & -\boldsymbol{X}_d & * & * & * & * & * & * & * \\ 0 & 0 & -\xi\tau\boldsymbol{I} & * & * & * & * & * & * \\ \boldsymbol{A}\boldsymbol{X}+\boldsymbol{B}\boldsymbol{Y} & \boldsymbol{A}_d\boldsymbol{X}_d & \xi\boldsymbol{E} & -\boldsymbol{X} & * & * & * & * & * \\ \boldsymbol{X} & 0 & 0 & 0 & -\left(d_s+1\right)^{-1}\boldsymbol{X}_d & * & * & * & * \\ \boldsymbol{Q}\boldsymbol{X} & 0 & 0 & 0 & 0 & -\xi\boldsymbol{Q} & * & * & * \\ \boldsymbol{R}\boldsymbol{Y} & 0 & 0 & 0 & 0 & 0 & -\xi\boldsymbol{R} & * & * \\ \boldsymbol{N}_A\boldsymbol{X}+\boldsymbol{N}_B\boldsymbol{Y} & \boldsymbol{N}_{Ad}\boldsymbol{X}_d & 0 & 0 & 0 & 0 & 0 & -\eta\boldsymbol{I} & * \\ 0 & 0 & 0 & \boldsymbol{M}^{\mathrm{T}} & 0 & 0 & 0 & 0 & -\eta^{-1}\boldsymbol{I} \end{bmatrix} < 0 \tag{S11}$$

Multiplying $\mathrm{diag}\{\boldsymbol{I}, \boldsymbol{I}, \boldsymbol{I}, \boldsymbol{I}, \boldsymbol{I}, \boldsymbol{I}, \boldsymbol{I}, \boldsymbol{I}, \eta\boldsymbol{I}\}$ on the both sides of the above inequality, (21) can be derived.

APPENDIX B

Proof: If $\frac{1}{\xi}\boldsymbol{x}^{\mathrm{T}}(k+1)\boldsymbol{P}\boldsymbol{x}(k+1) - \frac{1-\lambda}{\xi}\left(\gamma\boldsymbol{x}^{\mathrm{T}}\boldsymbol{P}\boldsymbol{x} + \gamma_d\boldsymbol{x}_d^{\mathrm{T}}\boldsymbol{P}\boldsymbol{x}_d\right) - \frac{\lambda}{\rho^2}\boldsymbol{p}^{\mathrm{T}}\boldsymbol{p} \leq 0$, then (27) can be guaranteed.

Substituting (S1) and $\boldsymbol{P} = \xi\boldsymbol{X}^{-1}$ to the above inequality, we can get:

$$\boldsymbol{\zeta}^{\mathrm{T}}(k)\,\mathrm{diag}\left(\gamma(\lambda-1)\boldsymbol{X}^{-1}, \gamma_d(\lambda-1)\boldsymbol{X}^{-1}, -\frac{\lambda}{\rho^2}\boldsymbol{I}\right)\boldsymbol{\zeta}(k) + \boldsymbol{\zeta}^{\mathrm{T}}(k)\boldsymbol{\Pi}_1^{\mathrm{T}}\boldsymbol{X}^{-1}\boldsymbol{\Pi}_1\boldsymbol{\zeta}(k) \leq 0 \tag{S12}$$

That means:

$$\mathrm{diag}\left(\gamma(\lambda-1)\boldsymbol{X}^{-1}, \gamma_d(\lambda-1)\boldsymbol{X}^{-1}, -\frac{\lambda}{\rho^2}\boldsymbol{I}\right) + \boldsymbol{\Pi}_1^{\mathrm{T}}\boldsymbol{X}^{-1}\boldsymbol{\Pi}_1 \leq 0 \tag{S13}$$

By using Schur complement, the following inequality can be obtained:

$$\begin{bmatrix} \gamma(\lambda-1)\boldsymbol{X}^{-1} & * & * & * \\ 0 & \gamma_d(\lambda-1)\boldsymbol{X}^{-1} & * & * \\ 0 & 0 & -\frac{\lambda}{\rho^2}\boldsymbol{I} & * \\ \boldsymbol{A}_C & \boldsymbol{A}_D & \boldsymbol{E} & -\boldsymbol{X} \end{bmatrix} \leq 0 \tag{S14}$$

Multiplying $\mathrm{diag}\{\boldsymbol{X},\boldsymbol{X},\boldsymbol{I},\boldsymbol{I}\}$ and its transpose from both sides of the above inequality.

$$\begin{bmatrix} \gamma(\lambda-1)\boldsymbol{X} & * & * & * \\ 0 & \gamma_d(\lambda-1)\boldsymbol{X} & * & * \\ 0 & 0 & -\frac{\lambda}{\rho^2}\boldsymbol{I} & * \\ \boldsymbol{A}_C\boldsymbol{X} & \boldsymbol{A}_D\boldsymbol{X} & \boldsymbol{E} & -\boldsymbol{X} \end{bmatrix} \leq 0 \tag{S15}$$

Substituting $\boldsymbol{A}_C=\boldsymbol{A}+\boldsymbol{B}\boldsymbol{K}+\boldsymbol{M}\boldsymbol{H}\left(\boldsymbol{N}_A+\boldsymbol{N}_B\boldsymbol{K}\right), \boldsymbol{A}_D=\boldsymbol{A}_d+\boldsymbol{M}\boldsymbol{H}\boldsymbol{N}_{Ad}$ and $\boldsymbol{Y}=\boldsymbol{K}\boldsymbol{X}$ to the above inequality, we can get:

$$\begin{bmatrix} \gamma(\lambda-1)\boldsymbol{X} & * & * & * \\ 0 & \gamma_d(\lambda-1)\boldsymbol{X} & * & * \\ 0 & 0 & -\frac{\lambda}{\rho^2}\boldsymbol{I} & * \\ 0 & \boldsymbol{A}_d\boldsymbol{X} & \boldsymbol{E} & -\boldsymbol{X} \end{bmatrix} + \begin{bmatrix} 0 \\ 0 \\ 0 \\ \boldsymbol{M} \end{bmatrix} \boldsymbol{H} \begin{bmatrix} \boldsymbol{N}_A\boldsymbol{X}+\boldsymbol{N}_B\boldsymbol{Y} & \boldsymbol{N}_{Ad}\boldsymbol{X} & 0 & 0 \end{bmatrix} + * \leq 0 \tag{S16}$$

According to Lemma 1, the above inequality holds if and only if there exists some positive scalar σ, such that:

$$\begin{bmatrix} \gamma(\lambda-1)\boldsymbol{X} & * & * & * & * & * \\ 0 & \gamma_d(\lambda-1)\boldsymbol{X} & * & * & * & * \\ 0 & 0 & -\frac{\lambda}{\rho^2}\boldsymbol{I} & * & * & * \\ \boldsymbol{A}\boldsymbol{X}+\boldsymbol{B}\boldsymbol{Y} & \boldsymbol{A}_d\boldsymbol{X} & \boldsymbol{E} & -\boldsymbol{X} & * & * \\ \boldsymbol{N}_A\boldsymbol{X}+\boldsymbol{N}_B\boldsymbol{Y} & \boldsymbol{N}_{Ad}\boldsymbol{X} & 0 & 0 & -\sigma\boldsymbol{I} & * \\ \boldsymbol{M}^{\mathrm{T}} & 0 & 0 & 0 & 0 & -\sigma^{-1}\boldsymbol{I} \end{bmatrix} \leq 0 \tag{S17}$$

Multiplying $\mathrm{diag}\{\boldsymbol{I},\boldsymbol{I},\boldsymbol{I},\boldsymbol{I},\boldsymbol{I},\eta\boldsymbol{I}\}$ on the both sides of the above inequality, (29) can be obtained. (30) guarantees the feedback control law $\boldsymbol{\delta}(k+i\mid k)=\boldsymbol{K}\boldsymbol{x}(k+i\mid k)$ satisfies the input constraint, the detail derivation process can be found in (Kothare et al., 1996).

APPENDIX C

Firstly, we need to proof the following recursive feasibility.

Recursive feasibility: If the optimization problem (31) is solvable at time k, it will always be feasible.

Proof: In optimization problem (31), only inequality (22) relates to the system state. Because (31) is solvable at time k, then $\boldsymbol{x}(k)$, $\boldsymbol{x}_d(k)\in\boldsymbol{\Omega}(k)$, where $\boldsymbol{\Omega}(k)$ denotes the set $\boldsymbol{\Omega}$ at time k. Because $\boldsymbol{\Omega}(k)$ is a RPI set, $\boldsymbol{x}(k+1)$ and $\boldsymbol{x}_d(k+1)$ also lie in the RPI set $\boldsymbol{\Omega}(k)$. Therefore, the optimization problem (31) is also feasible at time $k+1$. Through continuous iteration, recursive feasibility can be proofed.

The following is proof for Theorem 4.

Proof: We assume that $V_k^*(\boldsymbol{x}(k))$ is the optimal value of $V_k(\boldsymbol{x}(k))$ at time k. Define $V_k^*(\boldsymbol{x}(k))=\boldsymbol{\zeta}_3^{\mathrm{T}}(k)\boldsymbol{\Lambda}_3^*(k)\boldsymbol{\zeta}_3(k)$. Now we proof that $V_k^*(\boldsymbol{x}(k))$ is an ISS-Lyapunov function.

Define:

$$\rho_{\min}^*=\min\left\{\underline{\rho}\left(\boldsymbol{\Lambda}_3^*(k)\right)\right\}, \rho_{\max}^*=\max\left\{\bar{\rho}\left(\boldsymbol{\Lambda}_3^*(k)\right)\right\} \tag{S18}$$

where $\underline{\rho}(\cdot)$ and $\bar{\rho}(\cdot)$ denote the maximal and minimal eigenvalues separately. So, the following inequalities hold.

$$\rho_{\min}^{*} \left\|\boldsymbol{\zeta}_3(k)\right\|^2 \leq V_k^*(\boldsymbol{x}(k)) \leq \rho_{\max}^{*} \left\|\boldsymbol{\zeta}_3(k)\right\|^2 \tag{S19}$$

According to (19), we have

$$\begin{aligned} & V_k^*(\boldsymbol{x}(k+1)) - V_k^*(\boldsymbol{x}(k)) \leq -l(k \mid k) \\ & \leq -\boldsymbol{x}^{\mathrm{T}}(k)\boldsymbol{Q}\boldsymbol{x}(k) - \boldsymbol{\delta}^{\mathrm{T}}(k)\boldsymbol{R}\boldsymbol{\delta}(k) + \tau\boldsymbol{p}^{\mathrm{T}}(k)\boldsymbol{p}(k) \\ & < -\boldsymbol{x}^{\mathrm{T}}(k)\boldsymbol{Q}\boldsymbol{x}(k) + \tau\|\boldsymbol{p}(k)\| \\ & < -\boldsymbol{\zeta}_3^{\mathrm{T}}(k)\boldsymbol{Q}_{\boldsymbol{\zeta}_3}(k) + \tau\|\boldsymbol{p}(k)\| \end{aligned} \tag{S20}$$

$V_k^*(\boldsymbol{x}(k+1))$ is a possible selection of $V_{k+1}(\boldsymbol{x}(k+1))$. At time $k+1$, the optimal value of $V_{k+1}(\boldsymbol{x}(k+1))$ is $V_{k+1}^*(\boldsymbol{x}(k+1))$, then we have

$$V_{k+1}^*(\boldsymbol{x}(k+1)) \leq V_k^*(\boldsymbol{x}(k+1)) \tag{S21}$$

Then the following inequality can be obtained.

$$V_{k+1}^*(\boldsymbol{x}(k+1)) - V_k^*(\boldsymbol{x}(k)) < -\boldsymbol{\zeta}_3^{\mathrm{T}}(k)\boldsymbol{Q}_{\boldsymbol{\zeta}_3}(k) + \tau\|\boldsymbol{p}(k)\| \tag{S22}$$

According lemma 2, we complete the proof here.

REFERENCES

Kothare, M. V., Balakrishnan, V., and Morari, M. (1996). Robust constrained model predictive control using linear matrix inequalities. *Automatica* 32, 1361–1379
